# Th1‐type immune responses to Porphyromonas gingivalis antigens exacerbate angiotensin II‐dependent hypertension and vascular dysfunction

**DOI:** 10.1111/bph.14536

**Published:** 2018-12-26

**Authors:** Marta Czesnikiewicz‐Guzik, Ryszard Nosalski, Tomasz P Mikolajczyk, Francesca Vidler, Tomasz Dohnal, Elzbieta Dembowska, Delyth Graham, David G Harrison, Tomasz J Guzik

**Affiliations:** ^1^ Department of Periodontology and Oral Sciences Research Group, University of Glasgow Dental School and Institute of Infection, Immunity and Inflammation University of Glasgow Glasgow UK; ^2^ Department of Dental Prophylaxis and Experimental Dentistry Jagiellonian University School of Medicine Kraków Poland; ^3^ Institute of Cardiovascular and Medical Sciences University of Glasgow Glasgow UK; ^4^ Department of Periodontology Pomeranian Medical University Szczecin Poland; ^5^ Department of Clinical Pharmacology Vanderbilt University Nashville TN USA; ^6^ Department of Internal and Agricultural Medicine Jagiellonian University Medical College Kraków Poland

## Abstract

**Background and Purpose:**

Emerging evidence indicates that hypertension is mediated by immune mechanisms. We hypothesized that exposure to *Porphyromonas gingivalis* antigens, commonly encountered in periodontal disease, can enhance immune activation in hypertension and exacerbate the elevation in BP, vascular inflammation and vascular dysfunction.

**Experimental Approach:**

Th1 immune responses were elicited through immunizations using *P. gingivalis* lysate antigens (10 μg) conjugated with aluminium oxide (50 μg) and IL‐12 (1 μg). The hypertension and vascular endothelial dysfunction evoked by subpressor doses of angiotensin II (0.25 mg·kg^−1^·day^−1^) were studied, and vascular inflammation was quantified by flow cytometry and real‐time PCR.

**Key Results:**

Systemic T‐cell activation, a characteristic of hypertension, was exacerbated by *P*. *gingivalis* antigen stimulation. This translated into increased aortic vascular inflammation with enhanced leukocyte, in particular, T‐cell and macrophage infiltration. The expression of the Th1 cytokines, IFN‐γ and TNF‐α, and the transcription factor, *TBX21*, was increased in aortas of *P*. *gingivalis*/IL‐12/aluminium oxide‐immunized mice, while IL‐4 and TGF‐β were unchanged. These immune changes in mice with induced T‐helper‐type 1 immune responses were associated with an enhanced elevation of BP and endothelial dysfunction compared with control mice in response to 2 week infusion of a subpressor dose of angiotensin II.

**Conclusions and Implications:**

These results support the concept that Th1 immune responses induced by bacterial antigens such as *P*. *gingivalis* can increase sensitivity to subpressor pro‐hypertensive insults such as low‐dose angiotensin II, thus providing a mechanistic link between chronic infection, such as periodontitis, and hypertension.

**Linked Articles:**

This article is part of a themed section on Immune Targets in Hypertension. To view the other articles in this section visit http://onlinelibrary.wiley.com/doi/10.1111/bph.v176.12/issuetoc

**Abbreviations**
alum: aluminium oxide
Ang II: angiotensin II
*P. gingivalis*: *Porphyromonas gingivalis*
RT‐PCR: real‐time PCR
SNP: sodium nitroprusside
T_H_1: type 1 T‐helper cell
T_H_2: type 2 T‐helper cell
T_H_17: IL‐17‐producing CD4^+^ effector cell lineage

## Introduction

It has become increasingly evident that the immune system plays a critical role in the development of hypertension and its attendant end‐organ damage. Both adaptive and innate immune responses have been implicated in the pathogenesis of primary and secondary forms of hypertension (Harrison *et al*., 2011). In the 1960s, Okuda and Grollman proved that immunosuppression attenuates hypertension in rats (Okuda and Grollman, 1967). Similar BP‐lowering effects were observed upon treatment with anti‐thymocyte serum in hypertensive rats (Bendich *et al*., 1981). In 2007, we reported that RAG1‐deficient mice, which lack B and T cells have blunted hypertensive responses following the infusion of angiotensin II (Ang II). The development of vascular dysfunction in these mice is attenuated and their vascular production of free radicals in response to Ang II infusion is reduced (Guzik *et al*., 2007). T cells have been observed in the vessels and kidneys of humans with hypertension, and hypertensive humans have an increased number of circulating T cells that produce both type 1 T‐helper cell (T_H_1) and IL‐17‐producing CD4^+^ effector cell lineage (T_H_17) cytokines (Youn *et al*., 2013; Itani *et al*., 2016).

The mechanisms for T‐cell activation in hypertension and its effects on hypertension and vascular dysfunction remain incompletely understood (Meissner *et al*., 2017). Classically, T cells require antigen presentation by antigen‐presenting cells, and we have recently identified a role for isolevuglandin‐modified proteins as potential neoantigens in hypertension (Guzik and Channon, 2005; Kirabo *et al*., 2014). It is also possible that chronic infections, such as periodontitis, could lead to skewing of T‐cell populations and prime the host to exhibit enhanced immune responses to promote diseases such as hypertension. In hypertension, the immune responses have been shown to be skewed towards the Th1 type (Shao *et al*., 2003), but this has not yet been linked to vascular and BP phenotypes. Indeed, it has become recognized that the oral and gut microbiome can impact the immune system and that chronic localized inflammation can influence systemic health (Hansen *et al*., 2016; Regnault and Lacolley, 2017). Epidemiologically, periodontal disease represents one of most common examples of protracted inflammation. Inflammatory processes taking place in the gingivae and adjacent bone are initiated by the oral microbiome. In periodontitis, bacteria of the gingivae activate both innate and adaptive immunity and increase systemically circulating pro‐inflammatory cytokine levels and activated immune cells. Memory T cells formed in the setting of chronic infection can be activated to divide and proliferate in a heterologous fashion by non‐specific antigens and cytokines (Kim *et al*., 2002; Freeman *et al*., 2012). These cells could migrate to the vasculature and kidneys and alter vascular function, promote renal damage and contribute to hypertension. In line with this hypothesis, several studies show the coexistence of periodontal and cardiovascular disease in patients (Tsakos *et al*., 2010; Ricardo *et al*., 2015; Schmitt *et al*., 2015; Winning *et al*., 2015; Zeigler *et al*., 2015; Hansen *et al*., 2016). However, most of these studies suffer from clinical limitations related to confounding factors. Thus, direct evidence linking immune activation and hypertension is lacking. We therefore used an immunization model to study the role Th1 responses induced by antigens of the oral cavity, red complex bacteria *Porphyromonas gingivalis* antigens, in the development of Ang II‐dependent hypertension and vascular dysfunction. We chose to study *P*. *gingivalis* Th1 responses as previous studies, using the same system, have demonstrated a key role for these responses in periodontal pathology including bone loss and systemic inflammation (Stashenko *et al*., 2007; Leshem *et al*., 2008).

## Methods

### Animals and immunization protocol

Animal studies are reported in compliance with the ARRIVE guidelines (Kilkenny *et al*., 2010). C57BL/6J male (Jackson Laboratories, Bar Harbor, ME, USA), 12–14 weeks old, 25–30 g mice were immunized i.p. with *P*. *gingivalis* lysate reconstituted with aluminium oxide (alum) as an adjuvant and IL‐12 to skew T_H_1 cell‐mediated T‐cell responses. Immunizations were performed twice, 3 weeks apart. Sham mice were administered alum alone at the same time. Mice were randomly assigned to either the immunization or the alum‐only group. Individual mice were assigned numbers during randomization, and operators and data analysts for all subsequent endpoints were blinded for the treatment assignment groups. This model of immunization in C57BL/6J mice has been well‐characterized before in relation to a myriad of antigens, including *P*. *gingivalis* antigens (Goncalves *et al*., 2006; Stashenko *et al*., 2007; Leshem *et al*., 2008). Six days after the second i.p. immunization, osmotic minipumps (Alzet, model 2002, Cupertino, USA) were implanted s.c. under ketamine/xylazine anaesthesia [i.p. injection of ketamine/xylazine (100 mg·kg^−1^ / 10 mg·kg^−1^)] for infusion of a subpressor dose of Ang II, 0.25 mg·kg^−1^·day^−1^. The dose of Ang II was determined in a preliminary experiment (Supporting Information Figure S1). Ang II was administered for 14 days. During 14 days, Ang II infusion systolic BP was measured by tail‐cuff plethysmography (Hatteras MC 4000 – BP analysis system, Hatteras Instruments, Inc, Cary, USA) as previously described (Moore *et al*., 2015; Itani *et al*., 2016). The Emory University Institutional Animal Care approved all the experiments as the initial studies were performed at Emory University. Subsequent protocols were approved by the Jagiellonian University Institutional Animal Care Committee and Home Office Project Licence (led by Dr Delyth Graham) at the University of Glasgow. Mice were cared for in accordance with the Guide for the Care and Use of Laboratory Animals and housed five per cage in cages using standard bedding, fed *ad libitum* and maintained in the same room under a 12:12 h light/dark photoperiod at 22°C.

### P. gingivalis lysate formulation

*P*. *gingivalis* was chosen because it is a critical bacteria for the development of periodontitis (Rescala *et al*., 2010; Teles *et al*., 2010). The *P*. *gingivalis* lysate was a generous gift of Professor A. Campos‐Neto from Forsyth Institute, Boston, USA. The lysate was stored at −80°C. On the day of administration, the lysate was thawed, and 10 μg of *P*. *gingivalis* lysate was formulated with 50 μg of alum (Rehydragel HPA; Reheis, Berkeley Heights, NJ, USA). One microgram of recombinant mouse IL‐12 (Genetic Institute, Cambridge, MA, USA) was added prior to i.p. injections to additionally stimulate Th1 responses. In separate control experiments, IL‐12 (1 μg) alone was injected i.p. at the parallel time points to the ones used during immunizations.

### Flow cytometric analysis of leukocytes

After 14 days of Ang II administration, animals were killed, and flow cytometric analysis of leukocyte subsets in single‐cell suspensions of blood and the digested thoracic aorta was performed as previously described (Guzik *et al*., 2007).

For analysis of blood, leukocytes were isolated from the whole heparin‐treated blood after osmotic lysis of excess red blood cells. Cells were centrifuged (400× *g*), washed twice with PBS and 0.5% BSA (FACS buffer), counted, resuspended in 1% BSA/PBS and stored on ice for <30 min. Within 30 min, 10^6^ cells were stained for 15 min at 4°C with antibodies and washed twice with FACS buffer. Antibodies (all from Pharmingen, San Jose, CA, USA) used for staining in different multicolour combinations as follows: PerCP anti‐CD4, APC anti‐CD8, PE anti‐CCR5, FITC anti‐CD69, PE anti‐CD3. Mouse aortas were digested using collagenase type IX (125 U·mL^−1^), collagenase type IS (450 U·mL^−1^) and hyaluronidase I‐S (60 U·mL^−1^) dissolved in 20 mM HEPES‐PBS buffer containing calcium and magnesium for 30 min at 37°C, with constant agitation. Aortas were then passed through a 70 μm sterile cell strainer (Falcon; BD Biosciences, San Jose, CA, USA), yielding single‐cell suspensions. Cells were washed twice with 1% BSA/PBS buffer and additionally incubated for 30 min in 37°C with complete media (RPMI; 10% FCS), then washed again, counted and stained in different multicolour combinations as follows: FITC anti‐CD45, PerCP anti‐CD8, APC anti‐CD3, PE anti‐CD4, PerCP anti‐CD11b, APC anti‐CD11c, PE anti‐I‐A^b^, PerCP anti‐CD19, APC anti‐Gr1 and PE anti‐NK1.1. After the immunostaining procedure, cells were resuspended in FACS buffer and analysed immediately on an LSR‐II flow cytometer with DIVA software (Becton Dickinson Franklin Lakes, NJ, USA). Data were analysed with FlowJo software (Tree Star, Inc., Ashland, Oregon, USA). T cells were analysed as a percentage of the peripheral blood mononuclear cells (PBMCs) and also expressed in absolute numbers.

### Quantitative mRNA analysis

RNA was extracted using 1‐bromo‐3‐chloro‐propane (Sigma‐Aldrich Gillingham, UK) and reverse transcribed with oligo d(T)16 (Applied Biosystems, Branchburg, NJ, USA) according to the manufacturer's protocol. The cDNA served as a template for the amplification of genes of interest by real‐time PCR, using TaqMan Gene Expression Assays (*TBX21* – Mm00450960_m1, *GATA3* – Mm00484683_m1, IL‐4 – Mm00445259_m1, **TGFβ** – Mm00441724_m1, IFN‐γ – Mm00801778_m1 and TNF‐α – Mm00443258_m1) (all Applied Biosystems, Foster City, CA, USA), Universal PCR Master Mix (Applied Biosystems, Warrington, UK) and the 7900HT Fast Real‐Time PCR System (Applied Biosystems, Foster City, CA, USA). Target gene expression was calculated using the comparative method for relative quantitation upon normalization to 18S mRNA, and relative quantification was calculated as 2^−ΔΔCt^.

### Measurements of vascular reactivity

Relaxations to the endothelium‐dependent and ‐independent vasodilators **ACh** and **sodium nitroprusside (SNP)** were measured in isolated 3 to 4 mm segments of aorta in organ chambers as previously described (Guzik *et al*., 2007).

### Statistical analysis

Data in the article are presented as mean ± SEM. Data analysis and plot generation were performed with GraphPad Prism for MacOSX version 6.0c. To compare BP measurements in mice over time as well as vascular function studies, repeated measures ANOVA was used. Repeated measures ANOVA was chosen for examination of the same response over time (such as with BP or in response to an increasing dose of drug), as it reduces the impact of individual point variability and allows us to assess the difference as a whole. To compare numbers of T cells in aortas, Mann–Whitney *U*‐tests were performed. To show the effect of immunizations on hypertensive immune cell phenotype and aortic mRNA expression, we used one‐way ANOVA or Mann–Whitney *U*‐tests to analyse the flow cytometry data with Bonferroni correction. For comparison of the frequency of immune cell subsets, *χ* ^2^ analysis with Bonferroni correction was used. *P* values reported in the figures and tables represent the adjusted values after multiple testing. *P* < 0.05 was considered as statistically significant.

### Nomenclature of targets and ligands

Key protein targets and ligands in this article are hyperlinked to corresponding entries in http://www.guidetopharmacology.org, the common portal for data from the IUPHAR/BPS Guide to PHARMACOLOGY (Harding *et al*., 2018), and are permanently archived in the Concise Guide to PHARMACOLOGY 2017/18 (Alexander *et al*., 2017).

## Results

### Systemic T‐cell activation in the peripheral blood

**Figure 1:**
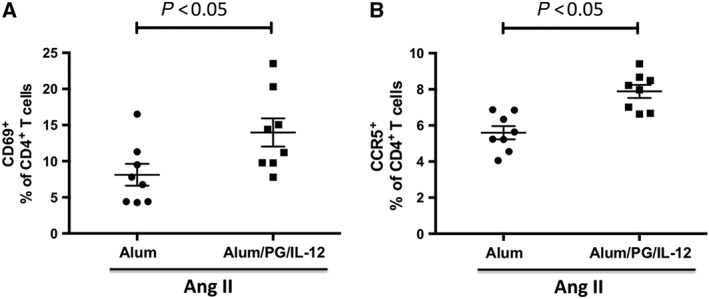
Systemic activation of peripheral blood T cells in immunized mice infused with a subpressor dose of Ang II. Mice were immunized with an i.p. injection of *P*. *gingivalis* formulated with alum and IL‐12 (Alum/PG/IL‐12) and the results are compared with control mice that received an i.p. injection of alum only (Alum). Six days after the second immunization, both groups received a 14 day infusion of a low dose of Ang II (0.25 mg·kg^−1^·day^−1^). Increased circulating CD4 T cells expressing CD69 (A) and CCR5 (B) in mice following i.p. injection of *P*. *gingivalis* formulated with alum and IL‐12 (Alum/PG/IL‐12) compared with control mice injected with alum alone (Alum) prior to a 14 day infusion of low dose of Ang II (0.25 mg·kg^−1^·day^−1^). CD69 and CCR5 were measured by flow cytometry in whole blood following red blood cell lysis as described in the Methods section. *P < 0.05 compared to control, *n* = 8 in each group.

Immunizations with *P*. *gingivalis* antigen, i.p., caused prolonged T‐cell activation, manifested by an increased proportion of circulating CD4^+^ T cells expressing the early activation marker CD69 and the chemokine receptor CCR5 when compared with control mice that received only i.p. alum (Figure 1A,B).

### Aortic inflammation is increased in Th1‐type‐immunized mice

**Figure 2:**
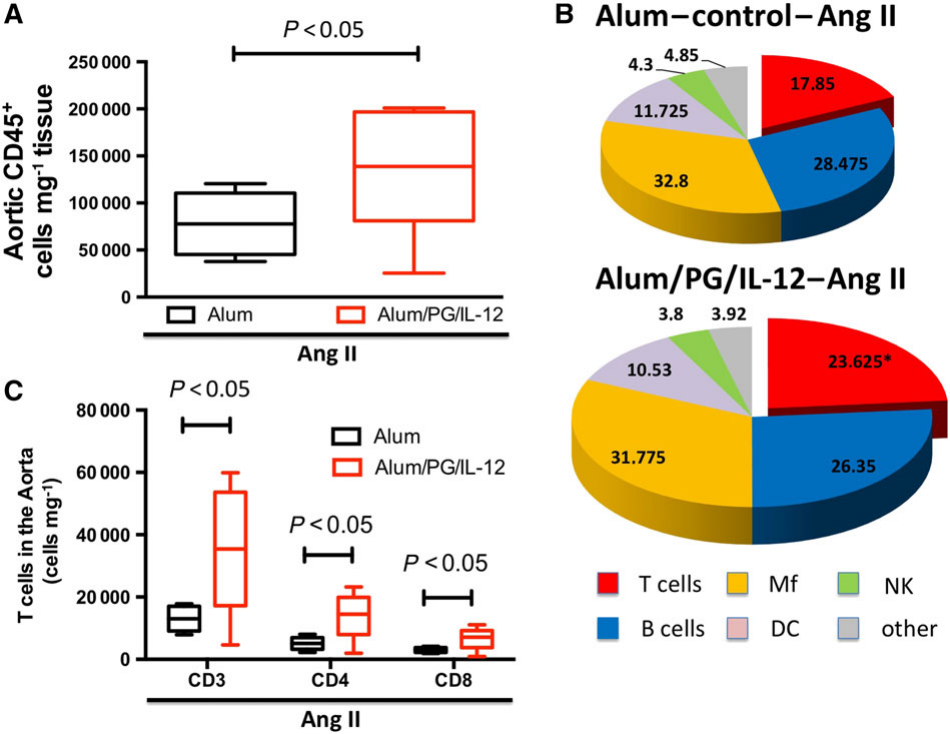
Characterization of aortic leukocytes following *P*. *gingivalis* immunization and low‐dose Ang II infusion. Mice were immunized with an i.p. injection of *P*. *gingivalis* formulated with alum and IL‐12 (Alum/PG/IL‐12) and are compared with control mice that received an i.p. injection of alum only (Alum). Following immunization, both groups received a 14 day infusion of a low dose of Ang II infusion (0.25 mg·kg^−1^·day^−1^). Flow cytometry was used to quantify total leukocytes (A, CD45^+^), *P < 0.05 compared to control, *n* = 8 in each group. Within the CD45^+^ cells, the percentage of T cells, B cells, macrophages, NK cells and dendritic cells were quantified (B). T cells – CD3^+^; lymphocytes B (B cells) – CD19^+^; macrophages (Mf) – I‐A^b+^/CD11b^+^; dendritic cells (DC) – I‐A^b+^/CD11c^+^; and NK cells (NK) – NK1.1^+^, *P < 0.05 versus control, *n* = 8 (C). T‐cell infiltration in aortas of *P. gingivalis*‐immunized mice and controls. T‐cell subpopulations (CD4^+^ and CD8^+^ T cells were studied) by flow cytometry following a 14 day infusion of a low‐dose of Ang II, *P < 0.05 compared to control, *n* = 8 in each group.

Flow cytometric analysis of single‐cell suspensions of aortas obtained from mice after 14 days of Ang II low‐dose infusion revealed that total aortic leukocytes, as reflected by CD45‐positive cells, were significantly increased in mice immunized with *P*. *gingivalis* compared with mice that received alum alone (Figure 2A). The composition of infiltrating immune cells was also altered in the *P*. *gingivalis* injected mice. These animals exhibited an increase in vascular T cells compared with mice that received alum alone. The percentage content of other major leukocyte subsets including B cells, macrophages, dendritic cells, NK cells and granulocytes remained unchanged (Figure 2B). Further characterization showed that the vascular content of both CD4 and CD8 subsets of lymphocytes were significantly increased (Figure 2C), although the increase in CD4^+^ cells was greatest.

**Figure 3:**
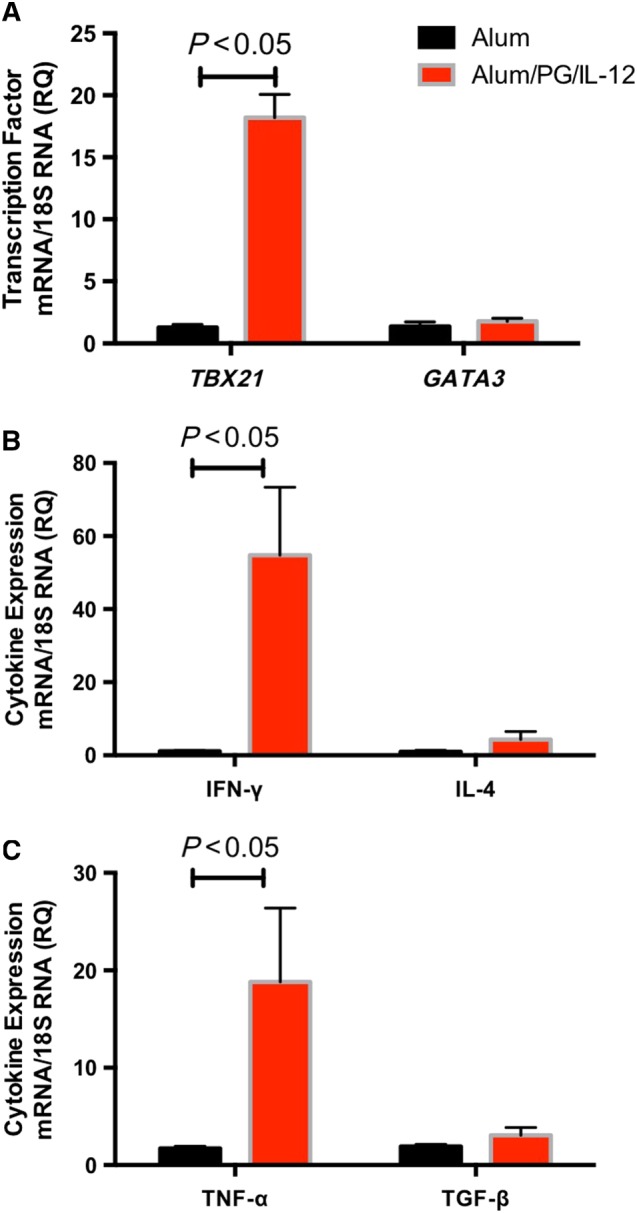
Characterization of Th1‐type inflammation following *P*. *gingivalis* immunizations and low‐dose Ang II infusion. Mice were immunized and treated with Ang II as in Figure 1. The aortic levels of mRNA for the transcription factor *TBX21* and *GATA3* (A), IFN‐γ and IL‐4 (B) and TNF‐α and TGF‐β (C) were measured using real‐time PCR. *P < 0.05 versus control, *n* = 6 in each group.

To further define vascular inflammation, we used RT‐PCR to measure mRNA levels of T‐cell cytokines and corresponding transcription factors after low‐dose Ang II infusion. In mice immunized with alum/PG/IL‐12, the levels of *TBX21* mRNA, a transcription factor characteristic of a T_H_1 immune response, were significantly increased, while mRNA for the type 2 T‐helper cell (T_H_2) transcription factor *GATA3* remained unchanged (Figure 3A). This was associated with increased aortic mRNA levels of IFN‐γ and TNF‐α, further characteristic of a T_H_1 response (Figure 3B,C) in the alum/PG/IL‐12 low Ang II mice compared with the alum control/low Ang II mice. Notably, very low mRNA levels of T_H_2 specific transcription factors or cytokines were detected.

### Blood pressure responses to low‐dose Ang II are exacerbated by Th1 immune activation by P. gingivalis antigens

**Figure 4:**
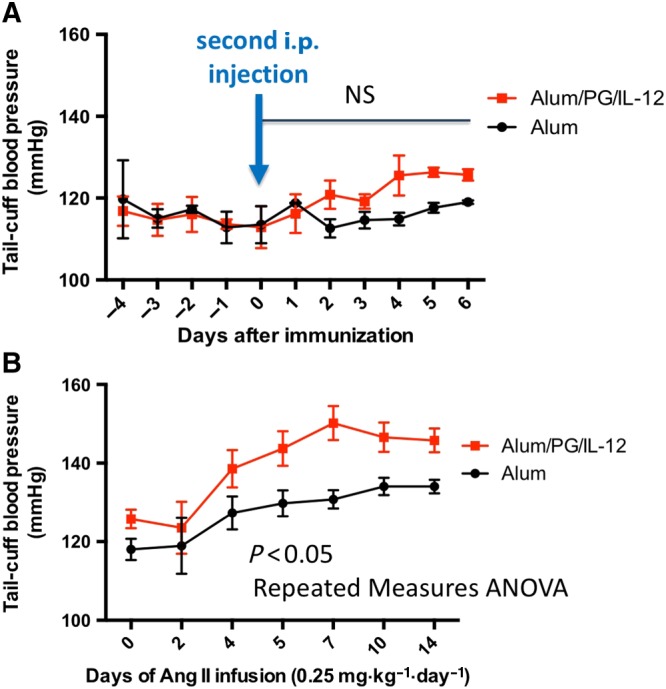
BP responses to low‐dose Ang II in mice immunized using *P*. *gingivalis* antigen (Alum/PG/IL‐12) and controls. Mice were immunized as in Figure 1. BP was measured daily before and after the second i.p. injection and before Ang II administration (A) and during a 14 day infusion of Ang II (0.25 mg·kg^−1^·day^−1^, B). *n* = 8 in each group. NS, not significant.

BP was monitored daily during alum/PG/IL‐12 immunization and alum injections. There was no difference in BP following the initial injection, and upon the second injection of alum/PG/IL‐12, there was a moderate but insignificant increase in BP when compared with alum alone (Figure 4A). Importantly, low‐dose Ang II administration caused a substantially greater increase in BP in alum/PG/IL‐12‐immunized mice than in alum control mice (Figure 4B).

### Endothelial dysfunction is exacerbated by Th1 immune activation by P. gingivalis antigens in hypertension

**Figure 5:**
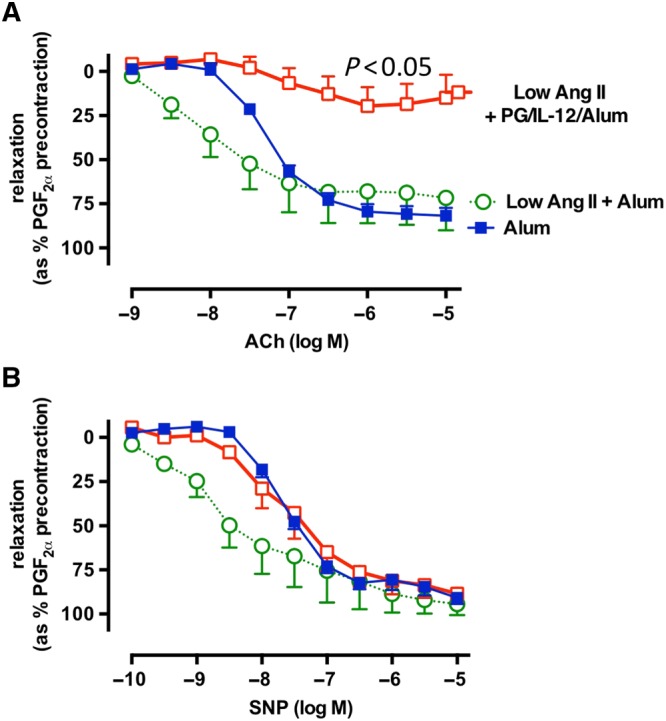
Effect of *P*. *gingivalis* immunization on endothelial function. Mice were immunized with an i.p. injection of *P*. *gingivalis* formulated with alum and IL‐12 (Alum/PG/IL‐12) and are compared with control mice that received an i.p. injection of alum only (Alum). Six days after the second immunization, both groups received a 14 day infusion of low dose of Ang II infusion (0.25 mg·kg^−1^·day^−1^). Isolated aortic segments were studied after a 14 day Ang II infusion in organ chambers. Following preconstriction with 3 × 10^−7^ M of PGF_2α_, vasodilatation to increasing concentrations of ACh (A) and SNP (B) was recorded and analysed by repeated measures ANOVA.

Endothelial dysfunction is a prominent determinant of hypertension; therefore, we next studied endothelium‐dependent vasodilatation in alum/PG/IL‐12 injected and alum control mice. Following 14 days of low‐dose Ang II, endothelium‐dependent vasodilatation was significantly impaired in the mice that had undergone prior immunization with alum/PG/IL‐12 compared with control mice that had received injections of alum alone (Figure 5A). In contrast, endothelium‐independent vasodilatation to SNP was not affected by alum/PG/IL‐12 (Figure 5B). Surprisingly, an increased sensitivity of blood vessels to low doses of ACh and SNP was observed in response to low dose of Ang II (Figure 5A,B). Importantly, IL‐12 injected alone did not have an effect on vascular function or BP (Supporting Information Figure S2).

## Discussion

In the present study, we demonstrated that the induction of Th1‐type responses to *P*. *gingivalis* antigens, one of the key oral bacteria responsible for periodontal disease, exacerbates the hypertensive response to Ang II, worsens vascular inflammation and impairs endothelial function. These data are valuable in the light of the ongoing discussion regarding the importance and potential mechanisms of immune activation that may play a modulatory role in hypertension. While we have not demonstrated specificity of these responses for *P*. *gingivalis*, we provide a proof of concept that is particularly valuable in light of the ongoing discussion about these associations between periodontal diseases and hypertension. Our study provides a mechanistic background and clear justification for subsequent investigations of the effects of oral periodontal infections on hypertension and vascular disease. Of relevance to the present findings, Th1‐type immune responses to similar immunization protocols have been shown to be essential for the development of periodontitis‐associated pathology, including bone loss (Stashenko *et al*., 2007).

Periodontal disease is a chronic inflammatory disease caused by oral microbiota, which leads to destruction of the soft tissue of the gingiva and bone of the alveolus. Epidemiologically, periodontal inflammation is probably the most common chronic inflammatory process in humans, affecting about half of Western populations (Patel, 2012; Tsukasaki *et al*., 2018). The junctional epithelium, which acts as a physiological zipper by binding the oral mucosa with the surface of the tooth, is destroyed in this process. Bacteria that subsequently invade the gingival tissue interact directly with the cells of the immune system and lead to immune activation. For this reason, we chose the *P*. *gingivalis* antigen as a stimulus for in the current studies. The demonstration of possible link between periodontal pathogens and hypertension is important in light of ongoing controversies regarding the relationships between periodontitis and hypertension. Ahn *et al*. (2015) showed a clear association between periodontal disease and hypertension in 14 625 women. Moreover, a recent meta‐analysis confirmed a relationship between periodontal diseases and high BP with an odds ratio of 1.15–1.67 (Martin‐Cabezas *et al*., 2016). This magnitude of risk is significant and is similar to the odds ratio of 1.51 for the association of obesity with hypertension. However, it is essential to note that this epidemiological evidence is based primarily on observational associations. The difficulty of establishing a cause–effect association between periodontal disease and hypertension is primarily linked to confounding factors. Both diseases have common risk factors including age, smoking, diabetes and poor nutrition. Another difficulty is related to the lack of unequivocal and uniform standards in diagnosis of periodontal disease across different studies (Grossi *et al*., 1996; Highfield, 2009). Some studies have included patients with severe periodontitis, while others have included moderate gingivitis in whom the junctional epithelium is intact and tissue invasion of microbiota is absent. This difficulty was emphasized in 2012, by an American Heart Association statement that although observational studies support an association between periodontitis and cardiovascular disease, there is insufficient evidence to unequivocally support causal relationship (Lockhart *et al*., 2012).

Our data provide new insight into the pathogenesis of hypertension. Classically, T cells require presentation of specific antigens to be activated. We have previously shown that T‐cell co‐stimulation is necessary for both Ang II and DOCA‐salt hypertension, supporting the idea that classical T‐cell activation *via* T‐cell ligation is needed. In keeping with the idea that specific antigens are presented in hypertension, we have found that isolevuglandin–protein adducts are presented by dendritic cells and can prime hypertension. We have also found that Ang II‐induced hypertension is associated with increased clonality of CD8^+^ T cells in the kidney, again suggesting a specific antigenic stimulus. In contrast, we have not observed clonality of T cells in the vasculature, despite an increase in the presence of both CD8^+^ and CD4^+^ T cells and most cells that accumulate in the kidney are not clonal. Of note, effector memory CD8^+^ cells, and to a lesser extent CD4^+^ T cells, are avidly recruited to localized sites of inflammation in an antigen‐independent fashion (Ely *et al*., 2003; McKinstry *et al*., 2010). In keeping with this, we found a striking increase in vascular CD4^+^ and CD8^+^ T cells in mice immunized with *P*. *gingivalis* and subsequently given Ang II. We have studied aorta, as it has been shown that periaortic inflammation contributes to endothelial dysfunction as well as vascular stiffening in these vessels. Therefore, studies of the aorta provide a good model reflecting a functional effect of immune activation in hypertension. These results support a ‘two‐hit’ hypothesis of hypertension development involving first a state of immune activation by stimuli like *P*. *gingivalis* and secondly a pro‐hypertensive effects of Ang II (Guzik *et al*., 2017).

Our study further emphasizes a role of T_H_1‐like cytokines including IFN‐γ and TNF‐α in hypertension. We have previously shown that immune clearing of TNF‐α blunts Ang II‐induced hypertension in mice (Guzik *et al*., 2007) and that IFN‐γ‐deficient mice likewise develop blunted hypertension during Ang II infusion (Kamat *et al*., 2015). In contrast, Garcia *et al*. (2012) have shown that IFN‐γ‐deficient mice develop more hypertrophy than wild‐type mice during aldosterone infusion but exhibit blunted hypertension to this stimulus. Recently, Sun *et al*. (2017) have shown that the mineralocorticoid receptor on T cells modulates IFN‐γ production and that specific deletion of this receptor markedly blunts experimental hypertension. IFN‐γ has been shown to enhance NADPH oxidase activity in macrophages and TNF‐α can stimulate superoxide production by vascular smooth muscle cells (De Keulenaer *et al*., 1998), thus could worsen hypertension *via* several related mechanisms (Cassatella *et al*., 1990). Another T_H_1 marker increased by our immunization strategy is CCR5, which interacts with the chemokine RANTES (CCL5). We have previously shown that the RANTES–CCR5 interaction plays an important role in activating T‐cell chemotaxis, adhesion and migration in hypertension (Mikolajczyk *et al*., 2016). Thus, the skewing of T_H_1 responses by our immunization protocol likely primed development of hypertension in response to the low dose of Ang II administered in these experiments. While our study was designed to test the effects of Th1 responses on BP and vascular dysfunction, it is important to emphasize that Th17 responses are known to play a critical role in hypertension and have also been associated with periodontitis (de Aquino *et al*., 2014; Souto *et al*., 2014). Our immunization protocol did not induce Th17 cells in alum/PG/IL‐12 mice (not shown). Indeed, IL‐12 is widely known to inhibit IL‐17 production (Hoeve *et al*., 2006), while inducing Th1 responses. Finally, in the present study, we did not measure circulating levels of cytokines. While this can be considered as a limitation, increased vascular infiltration of immune cells into the perivascular fat of immunized mice and increased Th1‐type cytokine expression may suggest the particular importance of the local inflammatory response.

Th1‐type‐inducing immunizations were associated with significant endothelial dysfunction, which could provide at least in part a mechanism for the exacerbated BP increases in this group of mice. It was intriguing that in our vasomotor experiments the sensitivity of blood vessels to low doses of ACh and SNP was increased in the presence of a low dose of Ang II. This may reflect increased VSMC responsiveness to NO that has been described in a number of disorders (Jebelovszki *et al*., 2008) and unquestionably would warrant further studies in the future. This is also interesting as inflammation is known to functionally modulate vascular smooth muscle cells (Allagnat *et al*., 2017; Fan *et al*., 2017).

It is important to note that we have used exclusively male mice in this study. This is related to the fact that it has been convincingly shown by several studies that female T cells are not participating in the pathogenesis of Ang II‐induced or salt‐sensitive hypertension in mice (Ji *et al*., 2014; Pollow *et al*., 2014).

While the search for hypertension‐specific antigens continues (Kirabo *et al*., 2014), one should not overlook the possibility that chronic inflammatory processes such as periodontal disease or obese adipose tissue inflammation (Akoumianakis and Antoniades, 2017) can contribute through pre‐activating T cells and monocytes and enable their migration to target organs (Barhoumi *et al*., 2017). This may reflect the involvement of both innate and adaptive immunity (Regnault and Lacolley, 2017; Zhang *et al*., 2017).

While the current studies provide proof of concept, there are limitations that should be acknowledged. The peritoneal mode of administration of the *P*. *gingivalis* antigen does not mimic the oral exposure encountered in periodontitis. An ideal model would involve oral infection; however, such approaches using oral gavage or a silk ligature to introduce the bacteria have not allowed either successful oral infection of mice or evidence for systemic activation and thus do not mimic the human disease. We would emphasize that our studies have only used *P*. *gingivalis* as a model antigen and that the main conclusion is that Th1 responses caused by antigens, including other pathogens, may likely lead to similar endpoints.

In summary, in the current study, we have provided proof of principle that Th1 responses induced by bacterial antigens such as *P*. *gingivalis* can increase sensitivity to subpressor pro‐hypertensive insult evoked by low‐dose Ang II. This supports the idea of ‘two‐hit’ hypothesis in which immune activation at sites of chronic inflammation exacerbates responses to otherwise minor stimuli such as low‐dose Ang II, therefore providing a link between chronic immune activation and hypertension.

## Author contributions

M.C.‐G. contributed to the development of the concept, in performing the experiments and in writing the manuscript; E.D. in the critical comments on the manuscript; D.G.H. in the development of the concept and in writing the manuscript; T.P.M. and R.N. in performing the experiments and in critical comments on the manuscript; F.V. in performing the experiments; D.G. in supervising the experiments; T.D. in the critical comments on the manuscript; and T.J.G. to the development of the concept, in performing and supervising the experiments and in writing the manuscript.

## Conflict of interest

The authors declare no conflicts of interest.

## Declaration of transparency and scientific rigour

This Declaration acknowledges that this paper adheres to the principles for transparent reporting and scientific rigour of preclinical research recommended by funding agencies, publishers and other organisations engaged with supporting research.

## Supporting information


**Figure S1** Titration experiment to precise the sub‐threshold dosage of Ang II which is still not elevating blood pressure alone in the experimental mice without any additional bacterial antigen exposure. Blood pressure was measured on the tail using tail‐cuff plethysmography (Hatteras MC 4000 ‐ blood pressure analysis system). Ang II dosage used: 0,25; 0,45; 0,7 mg·kg^−1^·day^−1^, *P < 0.05 *vs*. Pre‐Ang II; **P < 0.05 *vs*. 0,25 mg·kg^−1^·day^−1^, *n* = 8 in each group.

**Figure S2** Effect of IL‐12 intraperitoneal administration on development of vascular dysfunction (Panel A) upon low dose (0.25 mg·min^−1^·kg^−1^) Ang II administration and changes in systolic blood pressure (Panel B) upon two 1ug IL‐12 i.p. injections (21 days apart) followed by low dose (0.25 mg·min^−1^·kg^−1^) administration started 6 days after second immunization. Isolated aortic segments were studied after 14 day Ang II infusion in organ chambers as described before. Following pre‐constriction with 3x10‐7M of PGF2α, vasodilatation to increasing concentrations of acetylcholine (Panel A; ACh, left) and sodium nitroprusside (Panel A; SNP, right), were recorded and analysed by repeated measures ANOVA. IL‐12 administration effects on blood pressure at different stages of the experimental protocol were studied by tail cuff plethysmography. Data are expressed as normalized to no IL‐12 control for better visualization. *n* = 6 mice/group.

**Data S1** Supporting information.
