# Supplementary figures and images for: Th1‐type immune responses to Porphyromonas gingivalis antigens exacerbate angiotensin II‐dependent hypertension and vascular dysfunction
Source: Br J Pharmacol. 2018 Dec 26;176(12):1922–31. doi: 10.1111/bph.14536 (PMC6534780; doi:10.1111/bph.14536)

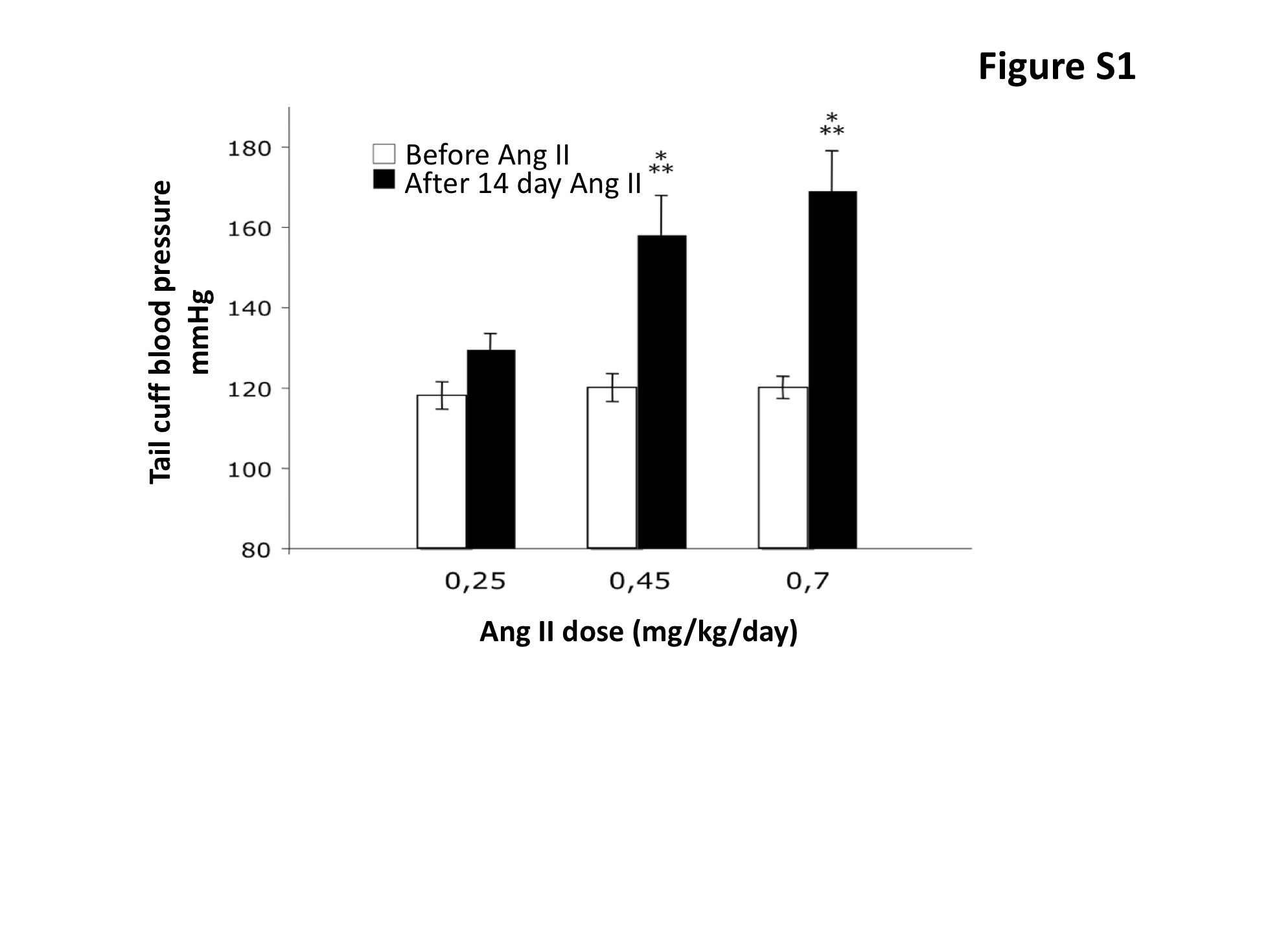

Supplement: Supplementary file 1 — Figure S1 Titration experiment to precise the sub‐threshold dosage of Ang II which is still not elevating blood pressure alone in the experimental mice without any additional bacterial antigen exposure. Blood pressure was measured on the tail using tail‐cuff plethysmography (Hatteras MC 4000 ‐ blood pressure analysis system). Ang II dosage used: 0,25; 0,45; 0,7 mg·kg−1·day−1, *P < 0.05 vs. Pre‐Ang II; **P < 0.05 vs. 0,25 mg·kg−1·day−1, n = 8 in each group. [file BPH-176-1922-s002.jpeg]

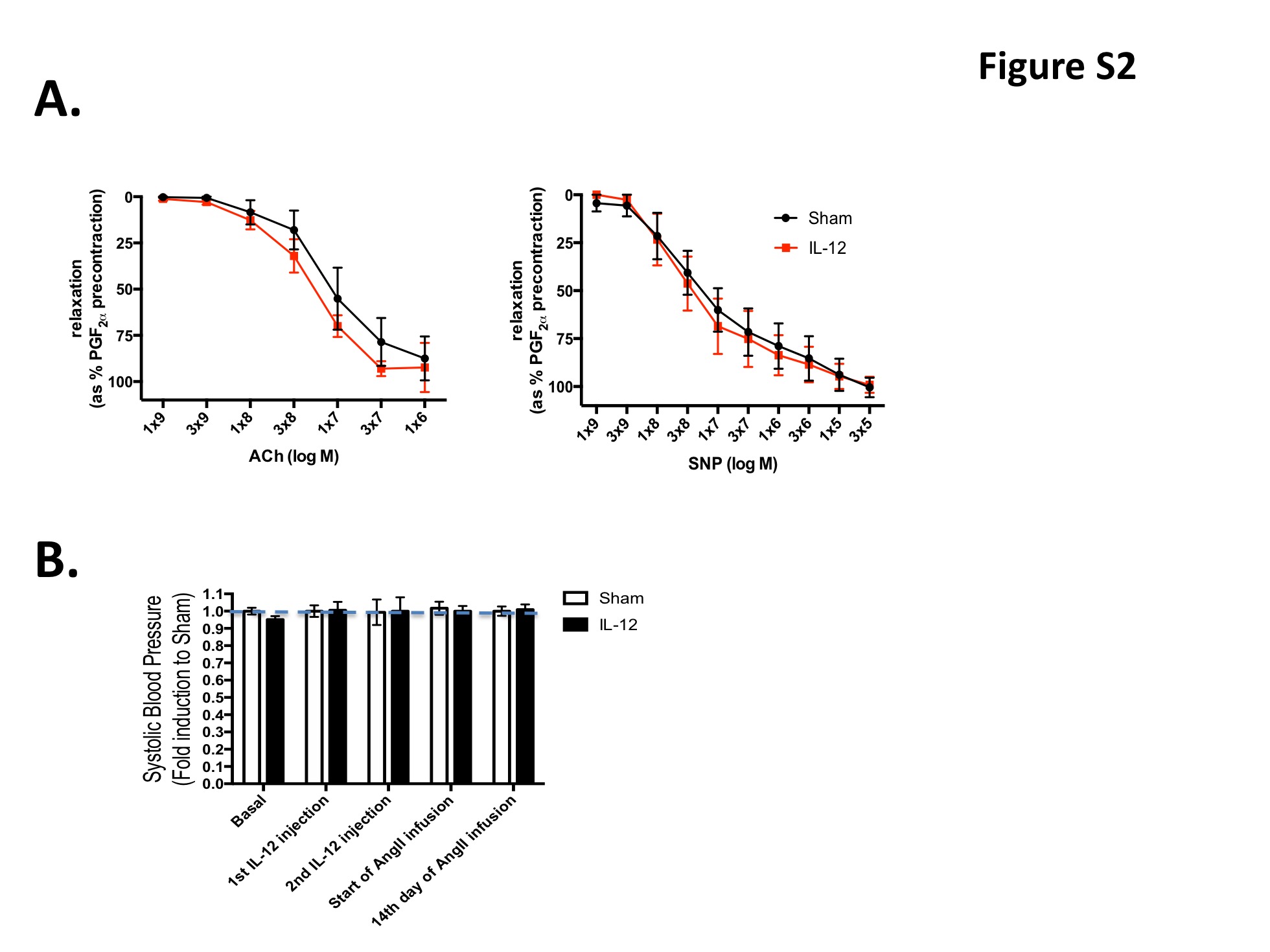

Supplement: Supplementary file 2 — Figure S2 Effect of IL‐12 intraperitoneal administration on development of vascular dysfunction (Panel A) upon low dose (0.25 mg·min−1·kg−1) Ang II administration and changes in systolic blood pressure (Panel B) upon two 1ug IL‐12 i.p. injections (21 days apart) followed by low dose (0.25 mg·min−1·kg−1) administration started 6 days after second immunization. Isolated aortic segments were studied after 14 day Ang II infusion in organ chambers as described before. Following pre‐constriction with 3x10‐7M of PGF2α, vasodilatation to increasing concentrations of acetylcholine (Panel A; ACh, left) and sodium nitroprusside (Panel A; SNP, right), were recorded and analysed by repeated measures ANOVA. IL‐12 administration effects on blood pressure at different stages of the experimental protocol were studied by tail cuff plethysmography. Data are expressed as normalized to no IL‐12 control for better visualization. n = 6 mice/group. [file BPH-176-1922-s003.jpeg]
