# Supplementary material for: Th1‐type immune responses to Porphyromonas gingivalis antigens exacerbate angiotensin II‐dependent hypertension and vascular dysfunction
Source: Br J Pharmacol. 2018 Dec 26;176(12):1922–31. doi: 10.1111/bph.14536 (PMC6534780; doi:10.1111/bph.14536)
Supplement: Supplementary file 3 — Data S1 Supporting information. [file BPH-176-1922-s001.pdf]

List of hyperlinks

Angiotensin II (Ang II)
CCL5
CCR5
IFN-γ
IL-12
IL-17
IL-4
TGF-β
TNF-α
